# The Negative Feedback-Loop between the Oncomir Mir-24-1 and Menin Modulates the Men1 Tumorigenesis by Mimicking the “Knudson’s Second Hit”

**DOI:** 10.1371/journal.pone.0039767

**Published:** 2012-06-27

**Authors:** Ettore Luzi, Francesca Marini, Francesca Giusti, Gianna Galli, Loredana Cavalli, Maria Luisa Brandi

**Affiliations:** Metabolic Bone Unit, Department of Internal Medicine, University of Florence, Florence, Italy; National Cancer Institute, National Institutes of Health, United States of America

## Abstract

Multiple endocrine neoplasia type 1 (MEN1) syndrome is a rare hereditary cancer disorder characterized by tumors of the parathyroids, of the neuroendocrine cells, of the gastro-entero-pancreatic tract, of the anterior pituitary, and by non-endocrine neoplasms and lesions. *MEN1* gene, a tumor suppressor gene, encodes menin protein. Loss of heterozygosity at 11q13 is typical of MEN1 tumors, in agreement with the Knudson’s two-hit hypothesis. *In silico* analysis with Target Scan, Miranda and Pictar-Vert softwares for the prediction of miRNA targets indicated miR-24-1 as capable to bind to the 3′UTR of MEN1 mRNA. We investigated this possibility by analysis of miR-24-1 expression profiles in parathyroid adenomatous tissues from *MEN1* gene mutation carriers, in their sporadic non-MEN1 counterparts, and in normal parathyroid tissue. Interestingly, the MEN1 tumorigenesis seems to be under the control of a “negative feedback loop” between miR-24-1 and menin protein, that mimics the second hit of Knudson’s hypothesis and that could buffer the effect of the stochastic factors that contribute to the onset and progression of this disease. Our data show an alternative way to MEN1 tumorigenesis and, probably, to the “two-hit dogma”. The functional significance of this regulatory mechanism in MEN1 tumorigenesis is also the basis for opening future developments of RNA antagomir(s)-based strategies in the *in vivo* control of tumorigenesis in *MEN1* carriers.

## Introduction

MicroRNAs (miRNAs) are a family of naturally occurring, evolutionary conserved, small (approximately 19–23 nucleotides), non-protein-coding RNAs that negatively regulate post-transcriptional gene expression. It is estimated that they account for >3% of all human genes and control expression of thousands of mRNAs, with multiple miRNAs targeting for a single mRNA [1]. Recent studies have also supported a role of miRNAs in the initiation and progression of human malignancies [2], as altered expression of miRNAs has been demonstrated in human tumors such as colorectal neoplasia [3], B cell chronic lymphocytic leukaemia [4], [5], B cell lymphoma [6], lung cancer [7], breast cancer [8], and glioblastoma [9], [10]. The involvement of miRNAs in human cancer is probably due to the fact that >50% of miRNA genes are located at chromosomal regions, such as fragile or common break point sites, and regions of deletion or amplification that are generally involved in tumorigenesis [11].

Multiple Endocrine Neoplasia type 1 (MEN1) syndrome is a rare complex tumor-predisposing disorder inherited in an autosomal dominant manner [12]. MEN1 syndrome is characterized by tumors of the parathyroids, of the neuroendocrine cells, of the gastro-entero-pancreatic tract, and of the anterior pituitary. *MEN1* gene, a tumour suppressor gene, whose translation product is the menin protein, is characterized by loss of heterozygosity at 11q13 in MEN1 tumors [12].

*In vitro* menin recognizes its mRNA and a specific RNA-protein-complex, also bound to MEN1 3′-UTR mRNA [13]. This suggests that the feedback oncosuppressor compensation by the wild type menin in *MEN1*-mutated cells could be regulated through RNA-protein-driven post-transcriptional mechanisms [13].

A ribonucleoprotein structure, in which multiple mRNAs are coordinately regulated by RNA binding proteins and by small non-coding RNA (RNA regulons) could be hypothesized in the menin-mRNA-miRNA(s)-transcription factors (Tfs) complex [14]. Several studies have demonstrated a central role of menin in the regulation of gene transcription. This regulatory effect can be both stimulatory and inhibitory [12]. Menin interacts with several proteins involved in transcriptional regulation and RNA expression analyses have identified several menin-regulated genes that could represent proximal or distal interaction sites for menin [15]. Tfs are essential regulators of gene expression, as they regulate transcription of target genes by specifically binding to the transcription factor binding site (TFBs) in gene promoter regions. The expression of a miRNA may be regulated by TF/TFs and miRNAs may regulate each other to form feed-back loops, or alternatively, both TFs and miRNAs may regulate their target genes and form feed-forward loops (FFLs) by creating a Gene Regulatory Network (GRN) [16].

*In silico* analysis with Target Scan, Miranda and Pictar-Vert softwares for the prediction of miRNA targets indicated miR-24-1 as capable to bind preferentially to the 3′UTR of MEN1 mRNA, and also to p27, p16, TGF-beta, and caspase 8, all involved in MEN1 tumorigenesis. In this work, analysis of miR-24-1 expression profiles performed in parathyroid endocrine tissues from *MEN1* mutation carriers, in their sporadic non-MEN1 counterparts and in normal parathyroid tissue, showed that the expression profiles of miR-24-1 mRNA and menin protein generate a GRN.

## Results

### An Evolutionary Conserved Target Sequence for miR-24-1 is Found in the 3′UTR of MEN1 mRNA

**Figure 1:**
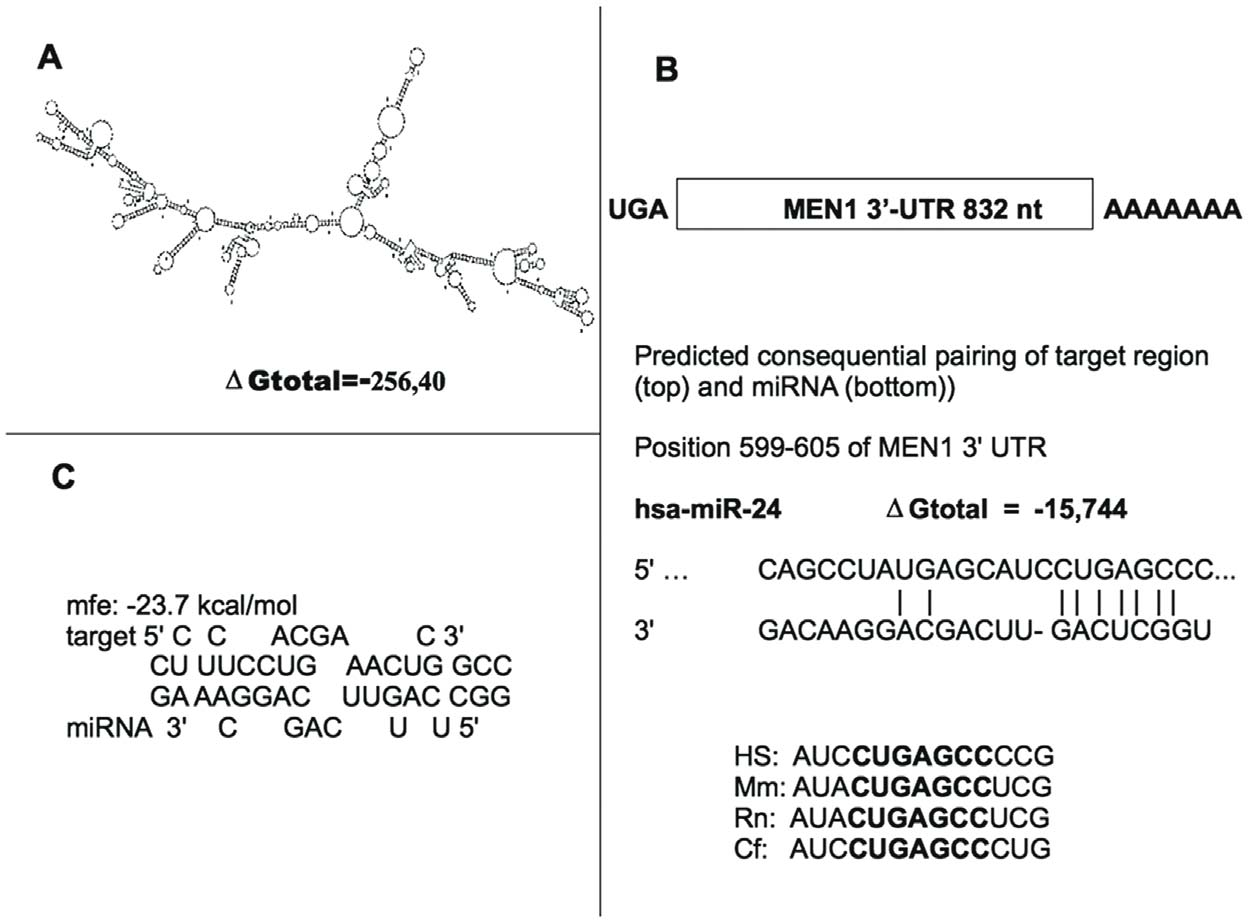
Putative miR-24-1 binding site on MEN1 3 ′**UTR mRNA.** Panel A: Predicted secondary structure of MEN1 3′UTR mRNA; Panel B: The location of the putative miR-24-1 on MEN1 3′UTR target site and the comparison of nucleotides between the miR-24-1 seed-sequence and its target in four species are shown; Panel C: Predicted hybridization of miR-24-1 and MEN1 3′-UTR by using RNAhybrid software. The minimum free energy (mfe) required for RNA hybridization is shown.

The highly structured 832 nt-3′UTR of MEN1 mRNA (Fig. 1A) was screened for complementarity to seed sequences of known miRNAs *via* a bioinformatic search by using TargetScan prediction (release 6.0) software. A 7mer-m8 seed match was found at nt 599–605 with a context score of 0.06 (Fig. 1B). This miRNA site was conserved in Human, Mouse, Rat, Dog, and Chicken (Fig. 1B). These data were confirmed by miRanda and PicTar algorithms as well. The minimum free energy (mfe) required for RNA hybridization is shown in Figure 1C. No nucleotide variation in the MEN1 3′UTR, that could affect the miR-24-1 binding, was found at positions 599–605 nt in the analyzed DNA samples.

### miR-24-1 Acts Directly at the MEN1 3′UTR

To confirm that miR-24-1 directly targets the highly-conserved sequence identified in the 3′UTR of MEN1 mRNA, a luciferase reporter assay was performed. 2′-*O*-methyl oligoribonucleotides were characterized as sequence-specific inhibitors of miRNA function and miRNA-direct RNA induced silencing complex (RISC) activity [1]. These molecules stoichiometrically bind and irreversibly inactivate miRNAs, providing a valuable tool to inhibit the function of a single miRNA both *in vitro* and *in vivo*.

**Figure 2:**
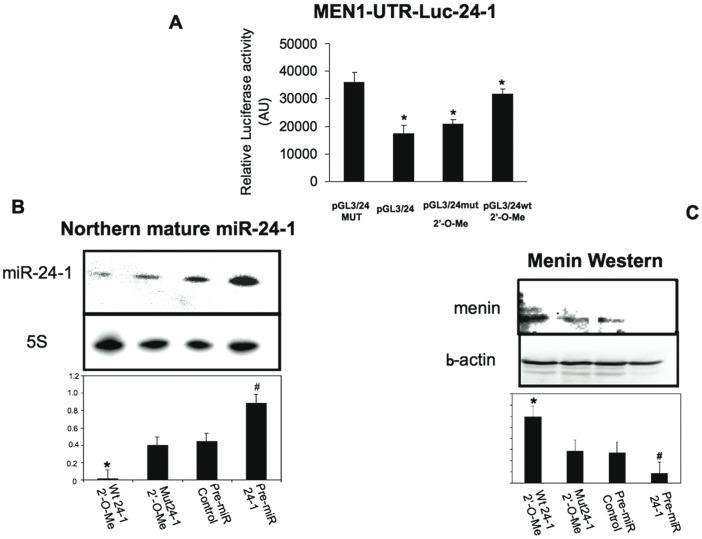
Luciferase assay and effects of miRNA inhibition/mimic. Panel A: WT reporter or mutated control luciferase plasmids were transfected into BON1 cells alone or with 2′-*O-*methyl miR-24-1 antisense RNA or 2′-*O-*methyl miR24-1 mutated antisense RNA (negative control). Reporter activities were measured after 2 days in differentiation media and normalized to *Renilla* luciferase activity. Bars indicate SD. *p<0.05 (paired Student’s *t*-test, *n = *3) with respect to the control negative sample. Panel B: Northern blot of mature miR-24-1 showing the selective inhibition of endogenous miR-24-,1 by specific miR-24 antisense RNA reported to the mutated antisense RNA as well as by the miR-24-1 overexpression with pre-miR-24-1 compared to the negative pre-miR-C. Bars indicate SD. * Wt 24-1 2′-O-Me vs Mut W24-1 2′-O-Me (*p<0.05, paired Student’s *t*-test, *n* = 3) and # Pre-miR24-1 vs Pre-miR Control (*p<0.05, paired Student’s *t*-test, *n* = 3). Panel C: Western blot analysis of menin expression performed to examine the effect of miR-24-1 on menin expression in BON1 cells treated with both 2′-*O-*methyl RNA and 2′-*O-*methyl pre-miR-mimic. An average of three experiments each performed in triplicate is presented. Bars indicate SD. *Wt 24-1 2′-O-Me vs Mut W24-1 2′-O-Me (*p<0.05, paired Student’s *t*-test, *n* = 3) and # Pre-miR24-1 vs Pre-miR Control (*p<0.05, paired Student’s *t*-test, *n* = 3).

To evaluate the degree of miRNA inhibition, the inhibition of miRNA-24-1 activity was verified by using pGL3/miR-24-1, a luciferase expression plasmid containing the complementary miR-24-1 binding sites. The luciferase expression plasmid containing the complementary miR-24-1 binding site (pGL3/24), and an analogous reporter with point substitutions disrupting the pairing to the miRNA seed of miR-24 acting as a negative control (pGL3/24MUT) were transfected in the human pancreatic neuroendocrine BON1 cells. The insertion of wild type sequences rendered the reporter sensitive to endogenous miR-24-1, with a reduction in luciferase activity when compared to the analogous mutated reporter (Fig. 2A). Thus, the effect of cellular endogenous miR-24-1 on translation of the luciferase mRNA was uniquely dependent on the presence of the miR-24-1 cognate binding site within the 3′UTR, as expression of the luciferase reporter containing the mutant miR-24-1 binding site within the 3′UTR was unaffected by the presence of endogenous cellular miR-24-1. Co-transfection of the reporter plasmid with 2′-*O*-methyl RNA antisense miR-24-1 (pGL3/24 wt+2′-*O*-Me) enhanced the expression of the reporter construct, indicating inhibition of endogenous miRNAs. By contrast, co-transfection of the reporter plasmid with control antisense RNA (pGL3/24+mut 2′-*O*-Me) did not have the same effect (Fig. 2A). This antisense-based-loss of function assay showed inhibition of endogenous miR-24-1 at a functional level.

The effect of miR**-**24-1 antisense 2′-*O*-methyl RNA and pre-miR-24-1 treatment on endogenous miR-24-1 and menin protein levels in BON1 cells was also determined. Pre-miR, the miRNA precursor molecules, are small, chemically modified double-stranded RNA molecules designed to mimic endogenous mature miRNAs. After transfection of these RNA oligonucleotides in the human pancreatic neuroendocrine BON1 cells, the endogenous expression levels of miR-24-1, and menin were analyzed respectively by Northern and by Western blot. Selective inhibition of endogenous miR-24-1, by specific miR-24-1 antisense oligonucleotides (Fig. 2B), induced an increase in menin expression (Fig-2C) when compared to the miR-24-1 mutated antisense oligonucleotides (Fig. 2B–C). Conversely, miR-24-1 reconstitution, by transfection with pre-miR-24-1 (Fig. 2 B), induced a reduction of menin expression (Fig. 2 C) due to the increased expression of mature miR-24-1 (Fig. 2 B).

### Inverse Correlation of Menin Protein and miR-24-1 Expression in MEN1 Parathyroid Adenomatous Tissues that Conserved the Wild-type Allele

**Figure 3:**
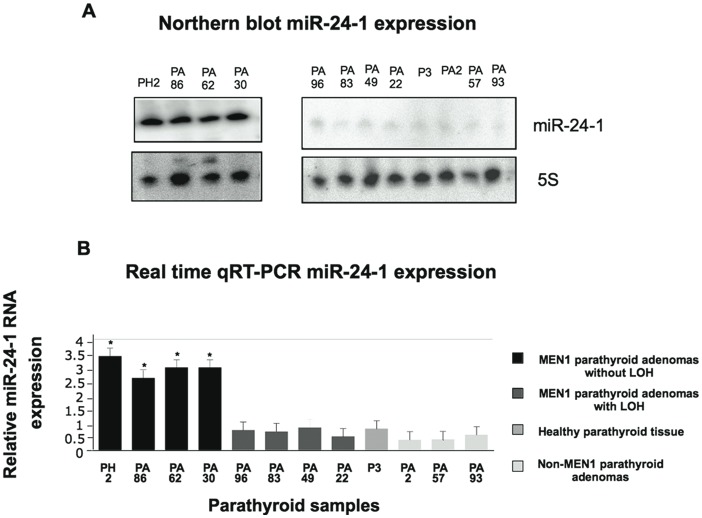
Expression analysis of mature miR-24-1 in parathyroid samples. Panel A: Northern blot analysis of mature miR-24-1 in normal parathyroid tissue, in MEN1 parathyroid adenomas and in non-MEN1 parathyroid adenomas; and panel B: Real time quantitative RT-PCR analysis of mature miR-24-1 in normal parathyroid tissue, MEN1 parathyroid adenomas, and non-MEN1 parathyroid adenomas. Bars indicate SD. An average of three experiments each performed in triplicate.* # *p*<0.05 (paired Student’s *t*-test, *n* = 3)with respect to the control sample, the normal parathyroid tissue.

If miR-24-1 was a negative tissue-specific regulator of the menin oncosuppressor protein, then miR-24-1 should be over-expressed in MEN1 parathyroid adenomatous tissues versus sporadic non-MEN1 forms of parathyroid adenomas as well as in healthy parathyroid tissue. Through the analysis of the different tissues for LOH at both the *MEN1* and *miR-24-1* loci, four MEN1 parathyroid tumors showed LOH for the *MEN1* allele (PA96, PA83, P49, and PA22), while four parathyroid tumors (PH2, PA86, PA62, and PA30) did not show *MEN1-*LOH, thus preserving the *MEN1* wild-type allele (Table 1). Both mutational and LOH analyses of *MEN1* allele in normal parathyroid and sporadic adenoma tissues showed homozygous wild-type *MEN1* allele. LOH analysis for *miR-24-1* gene did not show any LOH in any of the samples, as shown in Table 1. Northern blot analysis was performed to analyze the expression level of miR-24-1 in MEN1 parathyroid adenomas, in sporadic parathyroid adenomas and in one normal parathyroid tissue. As shown in Figure 3A, mature miR-24-1 was expressed only in MEN1 parathyroid adenoma tissues that conserved the wild-type allele (without *MEN1-*LOH), but not in the MEN1 parathyroid adenoma tissues that lost the *MEN1 gene* (with *MEN1-*LOH), as well as in sporadic parathyroid adenomas (Fig. 3 A). The different expression levels of mature miR-24-1, consistent with results of the Northern blot analysis, was confirmed by real-time qRT-PCR (Fig. 3B).

**Table 1:** Samples’ characteristics.

Pathology	*MEN1* mutation	Tissue code	LOH *MEN1* (11q13)	LOH *miR24-1* (9q22.32)
Normal parathyroid gland	NO	P3	NO	NO
Parathyroid adenoma (NON MEN1)	NO	PA2	NO	NO
Parathyroid adenoma (NON MEN1)	NO	PA57	NO	NO
Parathyroid adenoma (NON MEN1)	NO	PA93	NO	NO
Parathyroid hyperplasia (MEN1)	Frameshift, 1555insG, exon 10	PH2	NO	NO
Parathyroid adenoma (MEN1)	Frameshift, 953delGA, exon 6	PA30	NO	NO
Parathyroid adenoma (MEN1)	Frameshift, 317ins5(GCCCC), exon 2	PA62	NO	NO
Parathyroid adenoma (MEN1)	Frameshift, 359del4 (GTCT), exon 2	PA86	NO	NO
Parathyroid adenoma (MEN1)	Nonsense, Gln508Stop, exon 10	PA22	**YES**	NO
Parathyroid adenoma (MEN1)	Frameshift, 335delA, exon 2	PA49	**YES**	NO
Parathyroid adenoma (MEN1)	Nonsense, Gln508stop, exon 10	PA83	**YES**	NO
Parathyroid adenoma (MEN1)	Splicing 765-1(AG>AA), intron3-exon 4	PA96	**YES**	NO

### miR-24-1 is an Oncomir that Negatively Inhibits the Oncosuppressor Menin Protein After the First “Hit”

**Figure 4:**
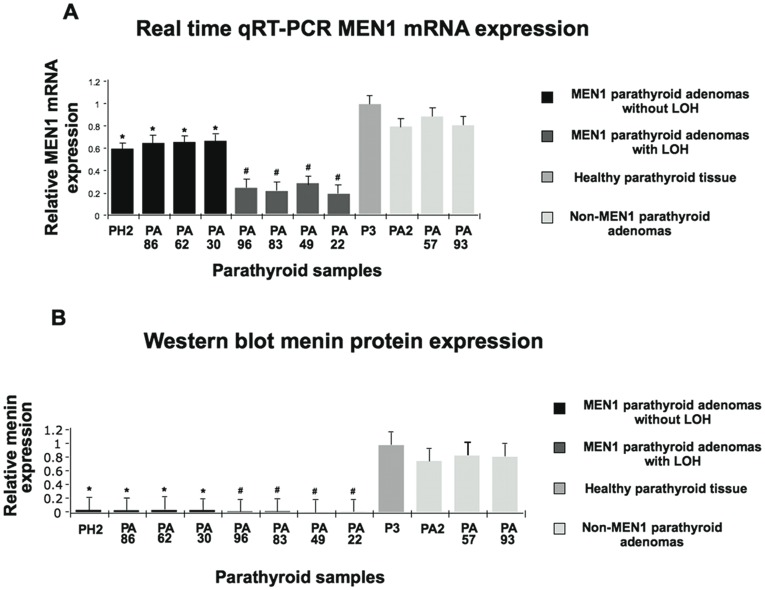
Expression analysis of menin protein and MEN1 mRNA. Panel A: Real time quantitative RT-PCR analysis of MEN1 mRNA in normal parathyroid tissue, in MEN1 parathyroid adenomas, and in non-MEN1 parathyroid adenomas. An average of three experiments each performed in triplicate; and panel B: Densitometric Western blot analysis (NIH-Image) in normal parathyroid tissue, in MEN1 parathyroid adenomas and in non-MEN1 parathyroid adenomas. Density ratio menin/beta-actin: bar diagram. Bars indicate SD. * # *p*<0.05 (paired Student’s *t*-test, *n* = 3) with respect to the control sample, the normal parathyroid tissue.

Those miRNAs whose expressions are increased in tumors may be considered oncogenes. These oncogene miRNAs are known as oncomirs and promote tumorigenesis by negatively inhibiting tumour suppressor genes. Indeed, miRNAs regulate gene expression through decreased translation, increased degradation of the target message, or both [4]. In order to check this hypothesis, we analyzed both the expression of MEN1 mRNA and menin protein in MEN1 and in sporadic parathyroid adenoma tissues. Real time qRT-PCR analysis of MEN1 mRNA expression showed that in MEN1 parathyroid adenomas that conserved one copy of the *MEN1* wild-type allele**,** there was around 50% reduction of MEN1 mRNA expression with respect to the control samples (normal parathyroid tissue and sporadic adenomas with the homozygous wild-type *MEN1* allele), while expression of MEN1 mRNA was strongly reduced in MEN1 parathyroid adenomas with LOH where wild-type *MEN1* gene was lost (Fig. 4A). Western blot in lysates from both MEN1 parathyroid adenomas with *MEN1-*LOH and without *MEN1-*LOH showed almost undetectable levels of menin expression with respect to the sporadic forms and the normal parathyroid tissue (Fig. 4B). These data are in agreement with the absence of MEN1 mRNA expression of MEN1-LOH parathyroid adenomas and high MEN1 mRNA expression levels in the sporadic parathyroid adenomas and in normal parathyroid tissue (Fig. 4A), but for the 50% expression level shown by MEN1 parathyroid adenomas that conserved one wild-type copy of *MEN1* gene (without *MEN1-*LOH), which should theoretically express half of the menin protein (Fig. 4A). These findings could be explained on the basis of a post-transcriptional negative control of MEN1 mRNA function in the presence of over-expressed miR-24-1 (Fig. 2A–B).

### Negative Regulatory Loop between miR-24-1 and Menin Exists in MEN1 Parathyroid Adenoma Tissues without LOH

**Figure 5:**
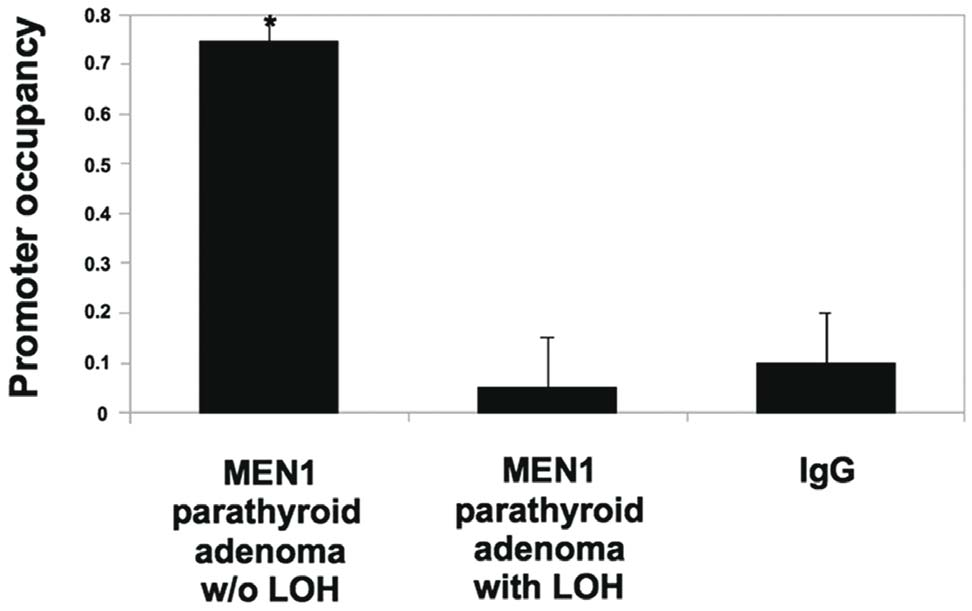
Chromatin immunoprecipitation analysis (ChIP). Chromatin extracts were prepared from a MEN1 parathyroid adenoma without LOH and a MEN1 parathyroid adenomas with LOH. Chromatin was immunoprecipitated with anti-menin antibody, or with IgG antibody as negative control. Quantitative SYBR green PCR was performed to determine whether the miR-24-1 promoter was present in the immunoprecipitate. Bars indicate SD. *p<0.05 (paired Student’s *t*-test, *n* = 3) with respect to the control sample.

Because regulatory feedback loops between miRNAs and their targets, mainly TFs, have been shown in several reports [17]–[19], we hypothesized that the data reported above indicated the possibility that, in MEN1 parathyroid adenoma tissues without LOH, miR-24-1 **and** menin are linked by a negative regulatory loop. To elucidate the transcriptional regulators involved in *miR-24-1* gene expression, we screened for transcriptional factor binding sites in miR-24-1. Menin-binding sites in the promoter of miR-24-1 were predicted by using the Transcription Element Search Software program (http://www.cbil.upenn.edu/cgi-bin/tess/tess), but we could not find any consensus menin binding sites. That is because, although menin is proven to be a TF [12], [15], there are no data in the database used by prediction softwares. To check this hypothesis, chromatin immunoprecipitation (ChIP) assay for menin was performed using parathyroid tissues from MEN1 patients. Co-precipitated DNA was analyzed by amplifying the genomic region containing miR-24-1 promoter region by real time PCR (Fig. 5). Occupancy of miR-24-1 promoter region was observed only in MEN1 parathyroid adenoma tissues without LOH (that conserved the wild-type copy of MEN1 allele), when compared to MEN1 parathyroid adenoma tissues with LOH and to control IgG (Fig. 5). These results indicate that menin occupies the miR-24-1 gene promoter, thus inducing its expression. As menin is a positive regulator of miR-24-1, while miR-24-1 negatively regulates the expression of menin, we conclude that menin and miR-24-1 form a “negative feedback loop” in the MEN1 parathyroid adenoma tissues without LOH (which thus still have one copy of the wild type allele).

## Discussion

In this study, we showed that in MEN1 syndrome, a rare complex tumor-predisposing disorder inherited in an autosomal dominant manner, the onset and progression of disease, after the first inherited “hit” is under the control of a “negative feedback loop” between menin and miR-24-1. This mechanism mimics the second somatic “hit” of tumor suppressor inactivation, thus indicating a potential mechanism for tissue-selective tumorigenesis in this hereditary tumor syndrome. Indeed, the main question regarding the pathogenetic basis of MEN1 syndrome is its predominant endocrine tumoral phenotype, with variable clinical manifestations not-related to the type of *MEN1* gene inactivating mutations. Candidate tissue-specific regulators may include miRNAs and TFs. We previously showed that in primary cultures of fibroblasts from MEN1 patients wild type *MEN1* gene allele-encoded mRNAs were expressed, whereas mutant alleles were partially degraded by a nonsense-mediated mRNA decay (NMD) pathway, suggesting a mechanism of feedback compensation for allelic loss by the up-regulation of wild-type menin expression at post-transcriptional level [13]. We also showed that menin forms a ribonucleoprotein structure in which multiple mRNAs should be coordinately regulated by RNA binding proteins and miRNAs [13]. This mechanism could be a way to control the haploinsufficiency that occurs when one allele is insufficient to confer the full functionality produced from two wild-type alleles.

In the present study, we analyzed the expression profile of miR-24-1 in parathyroid adenomas from MEN1 patients, with different *MEN1* mutations, in which the main difference was the *MEN1* LOH or the maintenance of the wild type MEN1 allele. Analysis of *miR-24-1* LOH showed no LOH for all the analyzed samples. While parathyroid adenomas from patients that lost both *MEN1* alleles showed no expression of MEN1 mRNA, of menin, or of miR-24-1, MEN1 parathyroid adenomas from patients that maintain one wild type copy of the *MEN1* gene showed, as expected, a reduced expression of MEN1 mRNA, but no expression of menin, while miR-24-1 was expressed. Thus, the induction of *miR-24-1* seems to depend on transcriptional expression of MEN1 mRNA. The ChIP study demonstrated the association of menin with the *miR-24-1* promoter, suggesting that the pri-miR-24-1 transcription was regulated by menin. The same mechanism does not seem to be involved in the sporadic parathyroid adenomas which usually occur later in the life of patients.

**Figure 6:**
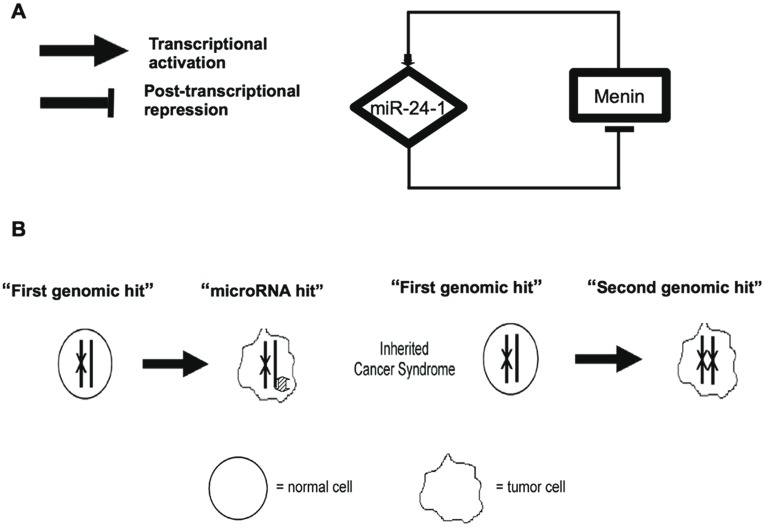
miR-24-1 menin Gene Regulatory Network. Panel A: Menin transcription activation regulates the transcription rate of miRNA 24-1 that repress post-transcriptionally menin. Panel B: Menin inactivation via “two hits”. The first “hit” is acquired in the germline with the second “hit” being acquired somatically.

**We showed that menin is the target of one miRNA**, the miR-24-1, that directly targets the highly-conserved sequence identified in the 3′UTR of MEN1 mRNA, thus offering an answer to the endocrine tissue-specificity of MEN1 tumors. Results from this study suggested that in MEN1 syndrome, after the first inherited germinal “hit”, the somatic onset and progression of neoplasia could be under the control of a “negative feedback loop” between menin protein and miR-24-1. We hypothesized that this regulatory network could mimic and substitute the second somatic “hit” of tumor suppressor inactivation in tissues in which MEN1 LOH has not yet occurred or could represent an intermediate step before the MEN1 LOH (Fig. 6). This mechanism should explain the proliferative changes in the neuroendocrine cells that could precede neoplasia. In the duodenum and the pancreas, the *MEN1* gene-associated germline mutation causes hyperplasia of gastrin, somatostatin and glucagon-secreting cells, resulting in multifocal development of tumors [20]. Indeed, these tumors show allelic deletion of the MEN1 gene, whereas the precursor lesions retain their heterozygosity [20]. In MEN1 the mechanism leading to neuroendocrine hyperplasia could be explained by the proposed “negative feedback loop” between menin and miR-24-1 acting as “homeostatic regulatory network” that need to be “broken” to induce the somatic “hit” and, consequently, to the neoplasia.

Many human genetic diseases result from loss-of-function germ-line mutations in one of the two homologous gene loci. These are often referred to as autosomal dominant diseases because of frequent phenotypic dominance of the mutated allele over the wild type allele during transmission along generations. There is presently one prevailing theory explaining the autosomal dominant expression of these diseases. This explanation originates from the Knudson two-hit theory of hereditary cancers, where loss of heterozygosity or occurrence of somatic mutations impairs the function of the wild-type copy [21]–[24]. While this somatic second hit may be sufficient for stable disease states, it is often difficult to determine if their occurrence necessarily marks the initiation of disease progression [25], [26]. Stochastic factors are likely to contribute to the characteristics of variable time of onset and incomplete penetrance of many autosomal dominant diseases [27], [28]. GRNs involving positive and negative feedbacks could control the effects of noise, by buffering its impact on gene expression [29]. In GNRs, TFs and miRNAs may reciprocally regulate one another to form “feedback loops”, or alternatively, both TFs and miRNA may regulate their target genes and form “feed-forward loops” [29]. Feedback loops are network structures that appear to have two functions in biological systems: they act as rapid switches to turn on a process [30], [31] and they act as noise buffers to enable a system to respond to long term signal changes [31]. Particularly, “negative feedback loops” potentially function to fine-tune gene expression and to maintain precise steady state levels of both components of the loop [29] and can result in oscillatory expression of both components, which depends on additional input signals [32]. We could hypothesize that the “negative feedback loop” between menin and miR-24-1 works buffering external and/or internal influences, as hormones and/or environmental factors. Epigenetic perturbations of this system could indeed drive the MEN1 tumorigenesis, as also supported by the individual clinical manifestation in MEN1 patients, independent on the type of *MEN1* gene mutation.

In conclusion, we showed that MEN1 syndrome could originate from mechanisms alternative to LOH. These events are under the control of a GRN, where miRNA-24-1 represents a strategic target to develop a therapeutic silencing antagomir to control MEN1 tumorigenesis progression [33].

## Materials and Methods

### Tissues Collection

We collected specimens from patients who underwent surgical parathyroid excision for benign adenomas; in particular we have obtained parathyroid tissues from MEN1 patients, exhibiting different *MEN1* gene mutations**,** as well as sporadic parathyroid adenoma samples from non-MEN1 patients (Table 1). Normal human parathyroid tissue used as healthy control was obtained by patients operated for thyroid carcinoma and pooled from three different cases. The data were analyzed anonymously and we used tissues collected before the establishment of “AOUC Ethics Committee”.

### Cell Cultures

Human pancreatic neuroendocrine BON1 tumour cells were kindly provided by Dr. Auernhammer (Munich, Germany). BON1 cells were cultured in DMEM/F12 (1:) medium (Life Technologies, Invitrogen, Foster City, CA USA) supplemented with 10% FCS., 1% penicillin/streptomycin (Life Technologies, Invitrogen), and 0,4% amphotericin (Biochrom, Cambridge, UK) in a 5% CO_2_ atmosphere.

### *MEN1* Gene Mutational Analysis

Genomic DNA of the MEN1 patients was extracted from peripheral blood leukocytes using a microvolume silica membrane-based column system according to the manufacture’s instructions (NucleoSpin Blood Quick Pure; Macherey-Nagel, Easton, PA, USA). Mutational analysis of the encoding regions (exons 2–10) and of the intron-exon junctions of the *MEN1* gene was performed by PCR-based direct sequencing.

### Loss of Heterozygosity Analysis

PCR-based microsatellite analysis for Loss of Heterozigosity (LOH) in the *MEN1* region was performed in parathyroid tissue DNA samples versus blood DNA samples. LOH analysis was performed using four specific microsatellite markers (D11S480, PYGM, D11S449, and D11S913) flanking the 11q12-13 locus. PCR-based microsatellite analysis for LOH in the 9q22.32 region, containing the human miR-24-1 gene, was performed in genomic DNA from parathyroid tissues versus blood-derived genomic DNA. LOH analysis was performed using 4 specific microsatellite markers (D9S167, D9S278, D9S283, D9S280) flanking the 9q22.32 locus. An independent PCR amplification was performed for each microsatellite in a final volume of 12.5 µl using PuReTaq Ready-To-Go PCR beads (GE Healthcare, Buckinghamshire, UK). An aliquot of each amplification product was denatured at 95°C for 5 min, in a solution of formamide and GENESCAN 400HD [ROX] size standard (Applied Biosystems), and then analyzed on the ABI Prism 3100 Genetic Analyzer (Applied Biosystems) by Genescan®analysis software.

### MicroRNA Target Prediction

The candidate targets of miR24-1 were identified based on a conservative intersection of the following microRNAs target prediction tools: TargetScan (http://genes.mit.edu/targetscan), Miranda (http://www.microrna.org/), and Pictar (http://pictar.bio.nyu.edu/). The minimum free energy required for RNA hybridization was predicted by using RNAhybrid software (http://bibiserv.techfak.uni-bielefeld.de/).

### *MEN1* 3′UTR Region Sequencing

The 3′UTR of the *MEN1* gene was analyzed, in blood-derived genomic DNA from all the subjects included in this study, by PCR-based direct and reverse sequencing for the screening of possible polymorphic variants, in the two binding sites to miR-24-1 evidenced by prediction tools (position 59–65 of *MEN1* 3′UTR and position 599–605 of *MEN1* 3′UTR), that could affect the miR-24-1-MEN1 mRNA bound.

### Oligoribonucleotides, Reporter Plasmids and Luciferase Assays

2′-*O*-methyl oligoribonucleotides miR-24-1 antisense, 5′-CUGUUCCUGCUGAACUGAGCC-3′; miR-24-1 antisense mutated, 5′-CUGUUCCUGCUGAACUGCUUU-3′) were synthesized by IBA (Gottingen, Germany). Reporter constructs that contain a miR-24-1 binding site (“pGL3-24-1”) or a mismatch sequence (“pGL3-24-1MUT”) in the 3′UTR of MEN1 mRNA were constructed by amplification of the full-length 3′UTR MEN1 (832 nt) using the following primers: 5′-CAGAGATCTAAGTCGTGTGAAATCATGTG-3′ (FW), 5′-TAAGTCGACAACAGGGTTTTCCAAGTCTA-3′ (RV) cloned into the Xba1-site of pGL3 (Promega, Madison, WI, USA), checked for orientation, sequenced and named.

Two hundred ng of Reporter or control plasmid (plus 80 ng pRL-null; Promega; Madison, WI, USA) were transfected, alone or in combination with 40 pmol of 2′-*O*-methyl oligoribonucleotides, in the BON1 cells, using Lipofectamine (Invitrogen). Luciferase assays were performed 48 h after transfection using the Dual Luciferase Reporter Assay System (Promega). Firefly luciferase activity was normalized to *renilla* luciferase activity for each transfected well. Each transfected well was assayed in triplicate.

### Transient Transfections

For the transfection experiments, a miR-24-1 precursor (pre miR-24-1) and a Pre-miR negative control (pre C; random sequence Pre-miR molecules extensively tested in human cell lines and tissues and validated to do not produce identifiable effects on known miRNAs function) were obtained by Ambion (Applied Biosystems). 2′-*O-*Me antisense miR-24-1 and miR-24-1 antisense mutated were described above. BON1 cells were transfected with RNAs (200 pmol) by using Lipofectamine 2000 (Invitrogen) according to the manufacturer’s instructions. Cells were harvested 48 h after initial transfection and proteins and RNAs were isolated by using “PARIS” (Ambion).

### Northern Blot Analysis

Total RNA was isolated with Trizol reagent (Invitrogen) according to the manufacturer’s instructions. For miRNA Northern blots, 15 µg of total RNA were separated on 15% denaturing polyacrylamide gels, electro-transferred to GeneScreen Plus membranes (PerkinElmer, Waltham, MA, USA), and hybridized using UltraHyb-Oligo buffer (Applied Biosystems) at 42°C overnight. Oligonucleotides, complementary to mature miRNAs, were ^32^P end-labeled with T4 Kinase (Roche Diagnostics, Indianapolis, IN, USA) and were used as probes. Probe sequences were: 5′-CTGTTCCTGCTGAACTGAGCCA-3′ as miR-24-1 antisense, and 5′-TTAGCTTCCGAGATCAGACGATTTTTCCTGTCTC-3′ as 5S antisense.

### Chromatin Immunoprecipitation

Chromatin immunoprecipitation (ChIP) was used to detect protein-DNA interactions. Tissues ChIP analysis was performed with a commercially available EpiQuik Tissue Chromatin Immunoprecipitation (ChIP) Kit in accordance with the manufacturer’s instructions. The chromatin fraction was immunoprecipitated overnight at 4°C using anti-menin (BL342) polyclonal antibody (Bethyl, Montgomery, TX, USA) and anti-IgG antibodies, followed by stringent washing and elution. Quantitative PCR amplification analysis was performed in a total volume of 25 ul with specific primers. The forward and reverse primers used for each gene were as it follows:

5′-AGCAGCTAGCAGGGTGATGT-3′; 5′-CATGGGAAGAACAGAGGATGA-3′.

### Quantitative Real-time PCR

Real-time RT-PCR was carried out as it follows. The experiment was replicated on two different cell samples. Ten µg of total RNA from each sample were DNAse treated with DNA-free kit (Ambion). Two µl of diluted RNA were reverse transcribed by using miScript reverse transcription Kit (Quiagen, Germany) and amplified using miScript SYBR Green PCR Kit and miR-24-1 and 5S miScript Primer assay (Quiagen) and MEN1 and 18S RNA Quantitec primer assay. Each RNA sample was evaluated for transcript levels in triplicate (including the normalization control 5S/18S) and quantified with MX3000P multiplex quantitative PCR instrument (Stratagene, La Jolla, CA, USA) following a unique three-step protocol: one cycle at 95° for 15 min and 40 amplification cycles (94°C for 15 sec, 1 min at 55°C and 70°C for 30 sec). Sample fluorescence was detected during the annealing step. The fluorescence data were collected continuously to obtain the dissociation curve. Fluorescence was plotted *versus* the Ct (threshold cycle) based on dRn (baseline-corrected, reference dye-normalized fluorescence) to obtain the standard curve and to measure the initial template quantity. Gene expression was normalized to *5S RNA or 18S RNA.* Fluorescence data were analysed using MXPro Software.

### Western Blotting

Cell and tissue lysates were prepared by the PARIS purification kit. Protein concentrations were determined by the BCA protein assay (Pierce, Rockford, IL, USA) using bovine serum albumin as the standard protein. Fifteen µg of each cell protein extract were denatured 10 minutes at 95°C in a volume of 95% Laemmli Sample Buffer (Bio-Rad, Hercules, CA, USA) and 5% beta-mercaptoethanol solution. Proteins were separated by SDS-12% polyacrylamide gel electrophoresis. The separated protein bands were transferred electrophoretically to a nitrocellulose membrane (Optitran BA-S 83, Schleicher & Schuell, Dassel, Germany) for 1 hour in a Mini Trans-blot electrophoretic transfer cell (Bio-Rad) at 100 V. Filters were blocked for 1 hour at room temperature in a blocking solution of PBS, 0.1% tween 20 and 2% ECL Advance blocking agent (Amersham Biosciences, Little Chalfont, UK). To evaluate menin expression, the blocked membrane was incubated with rabbit anti-menin (BL342) polyclonal antibody (Bethyl) for 1 hour at room temperature (1∶1000 dilution of stock in blocking solution), washed 4 times for 5 minutes each with a solution of PBS and 1% tween 20, incubated with an antirabbit horseradish peroxidase-conjugated secondary antibody (Santa Cruz, CA, USA) for 1 hour at room temperature (1∶2000 dilution of stock in blocking solution), then washed 4 times for 5 minutes each with a solution of PBS and 1% tween 20 and finally washed once with PBS. Detections of membrane-bound anti-menin antibody were performed by chemiluminescence. Beta-actin was used as a housekeeping protein.

### Statistical Analysis

Data are expressed as mean ± SD. All blots represent at least three separate experiments. Data shown were done in triplicate for each experiment, and are representative of three or more experiments. Comparisons were made by using a two-tailed *t* test or one-way ANOVA for experiments with more than two subgroups. Probability values were considered statistically significant at p<*0.05.* **Due to the rarity of MEN1 syndrome, the current parathyroid sample size is relatively small, thus the potency of the current statistical analysis could be reduced.**
